# Postoperative morbidity and mortality from aortic valve replacements in 25 cases in Senegal

**DOI:** 10.11604/pamj.2020.36.118.24000

**Published:** 2020-06-23

**Authors:** Momar Sokhna Diop, Papa Salmane Ba, Abdoulaye Boubou Aw, Papa Amath Diagne, Ndeye Fatou Sow, Papa Ousmane Ba, Amadou Gabriel Ciss

**Affiliations:** 1 Department of Thoracic and Cardiovascular Surgery, Cheikh Anta Diop University, Dakar, Senegal

**Keywords:** Aortic valve replacement, morbidity, mortality, Senegal

## Abstract

The purpose is to study the short- and medium-term morbidity and mortality linked to the implantation of an aortic prosthesis during cardiac surgery. This is a longitudinal, retrospective and descriptive study which takes place over a period from January 2017 to March 2020 (38 months) at the level of the thoracic and cardiovascular surgery clinic of the university Hospital Center of Fann in Dakar. All patients who underwent aortic valve replacement during this period were included in the study. A number of the series was 25 patients with a sex ratio of 2.66. The average age of the patients was 29.5 years (8-51 years). In the patients’ history, 19 patients (76%) had a notion of recurrent angina. Exercise dyspnea was the most common functional symptomatology present in 24 patients (96%). In the series, there were 22 cases (88%) of aortic insufficiency of various grades (2 to 4) with 7 cases (28%) associated with mitral insufficiency. We had 3 cases (12%) of aortic stenosis. All patients received surgical management under cardiopulmonary bypass. The average duration of cardiopulmonary bypass was 132 minutes ± 41.21 (53-226 minutes). The average duration of aortic clamping was 101 minutes ± 31.87 (53-164 minutes). The surgical procedures consisted in replacing the aortic valve with a biological prosthesis in one patient (4%) and a mechanical prosthesis in 24 patients (96%). The average length of hospital stay in intensive care was 5 days ± 4.03 (2-20 days). The average length of hospital stay was 20.76 days ± 13.19 (9 to 64 days). The average duration of follow-up was 8.2 months ± 4.57 (1 week - 32 months). During the follow-up, only one patient (4%) had developed infectious endocarditis on prosthesis and only one patient (4%) had a complication related to anticoagulant therapy (antivitamin K) such as gingivorrhagia and melena. We had recorded a single case of death at 6 months, a late mortality of 4%. Aortic valve replacement surgery, by median sternotomy gives satisfactory short- and medium-term results with negligible morbidity and negligible operative mortality.

## Introduction

Aortic valve disease is any damage to the aortic valve. The infringement may consist of regurgitation, narrowing or the combination of the two defining aortic valve disease. The causes of aortic valve disease are diverse and are dominated in Africa by rheumatic origin [1]. Aortic valve replacement surgery is one of the therapeutic strategies in the management of these pathologies guaranteeing a better survival and quality of life for the patient [2]. It has become commonplace these days with huge progress and very good results, but it still has many complications related to the underlying heart condition and the cardiopulmonary bypass. Advances in surgical techniques, myocardial protection and post-operative resuscitation have greatly reduced the mortality and morbidity associated to this surgery.

## Methods

Our study is longitudinal, retrospective and descriptive. It takes place in Dakar over a period from January 2017 to March 2020, i.e. a duration of 38 months. The data come from patient records operated for aortic valve disease at the thoracic and cardiovascular surgery clinic of Fann University and Hospital Center in Dakar. All patients who underwent aortic valve replacement during this period were included in the study. The data were collected on a pre-established sheet. They were captured with the Microsoft Excel software 2013. The descriptive study was carried out with the calculation of frequencies and proportions for the qualitative variables and the calculation of the means for the quantitative variables.

## Results

**Figure 1:**
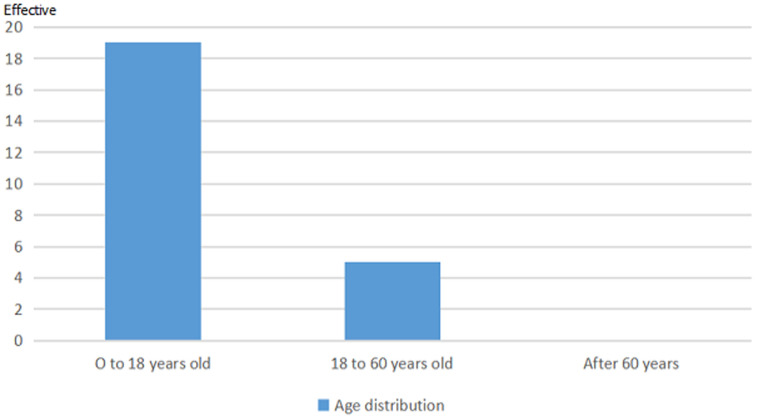
age distribution of patients

The total number of patients of our study was 25 (18 men and 7 women). There was a male predominance with a sex ratio of 2.66. The average age of the patients was 29.5 years (8-51 years) (Figure 1). In the patients’ history, 19(76%) had a concept of recurrent angina and 10 patients (40%) had a concept of cardiac decompensation. Exercise dyspnea was the most common functional symptomatology in 24 patients (96%). Chest radiography found cardiomegaly in 24 patients (96%) with signs of pulmonary hypertension in 18 cases (72%). On the electrocardiogram, we have found atrial fibrillation in 2 cases (8%). The preoperative echocardiographic data are summarized in the following table (Table 1). In the series, 22 cases were noted, that is 88% of aortic insufficiency of various grades (2 to 4) with 7 cases (28%) associated with mitral insufficiency. We had 3 cases (12%) of aortic stenosis. All patients received surgical management under cardiopulmonary bypass. The approach was a vertical midline sternotomy in all patients. The average duration of cardiopulmonary bypass was 132 minutes ± 41.21 (53-226 minutes). The average duration of aortic clamping was 101 minutes ± 31.87 (53-164 minutes). Cardioplegia was crystalloid in 32% of cases and blood in 68% of cases injected anterograde directly into the coronary ostia.

**Table 1:** preoperative echocardiographic parameters

Valvular heart disease	Parameters	Mean	Standard deviation	Minimum	Maximum
Aortic regurgitation (N=22 cases)	VC (mm)	9.84	12.09	3.7	34
	ERO (mm^2^)	66	48.68	9	179
	RV (ml)	120.17	85.14	20	340
Aortic stenosis (N=3 cases)	Aortic area (cm^2^)	0.6	0.21	0.4	0.82
	Mean gradient (mmHg)	32.7	27.01	8.5	88
	Systolic pulmonary arterial pressure (mmHg)	34	7.74	24	47
Left ventricular ejection fraction	%	64	6.87	51	80.8

N: Number; VC: Vena Contracta; ERO: Effective Regurgitant Orifice; RV: Regurgitant Volume; mmHg: millimeter of mercury; mm: millimeter; cm2: Centimeter square; ml: milliliter

The surgical procedures consisted in replacing the aortic valve with a biological prosthesis type Braile Biomedica No. 21 in a single patient (4%) and a mechanical prosthesis in 24 patients (96%) distributed as follows (Table 2). Seven patients (28%) underwent a mitral repair and 3 (12%) had an associated tricuspid repair. The average length of stay at the hospital in intensive care unit was 5 days ± 4.03 (2-20 days). The average duration of intubation was 5 hours ± 3.53 (2-16 hours). In our series, 11 patients (44%) showed hemodynamic instability in intensive care unit, 6 patients (24%) arrhythmia, 3 patients (12%) conduction disorders, 7 patients (28%) an inflammatory systemic response syndrome (SIRS), only 1 patient (4%) had developed an infection of the operating site and 6 patients had developed a malaria attack. The postoperative control echocardiographic parameters are summarized in Table 3. No patient had a prosthetic thrombosis. The average length of stay at the hospital was 20.76 days ± 13.19 (9 to 64 days). The average duration of follow-up was 8.2 months ± 4.57 (1 week - 32 months). During the follow-up, only one patient (4%) had developed infectious endocarditis on a prosthesis and only one patient (4%) had a complication related to anticoagulant therapy (antivitamin K) such as gingivorrhagia and melena. We found conduction disorders in the type of 1^st^ degree atrioventricular block in 3 patients. We have recorded a single case of death occurred at 6 months, a late mortality of 4%.

**Table 2:** characteristics of mechanical prostheses

Prosthesis type	Number	Size
ATS	1	24
Carbomedics	7	16, 21, 23
St Jude	16	19, 20, 21, 22, 23 et 25

**Table 3:** postoperative echocardiographic parameters

Parameters	Mean	Standard deviation	Minimum	Maximum
Left ventricular ejection fraction (%)	62	10.49	50	78
Systolic pulmonary arterial pressure(mmHg)	26.7	7.70	17	45
Medium gradient (mmHg)	15.66	9.97	2.15	36
TAPSE (mm)	17.11	4.24	11.6	24

mmHg: millimeter of mercury; TAPSE: Tricuspid Annular Plane Systolic Excursion; mm: millimeter

## Discussion

The etiologies of aortic valve disease are dominated in Africa by rheumatic fever. Valvular infringement consists of insufficiency, aortic stenosis or the combination of the two mechanisms. When feasible, aortic repair is most often preferred than valve replacement, especially in children. However, this repair is still not possible and valve replacement becomes necessary. This method presents some difficulties, particularly in children and young adults because of the size and the choice of prostheses available [3-5]. In our series the average age was 29.5 years (8-51years) and 76% of the patients were under 18 years of age. On the other hand, the use of homografts or a biological prosthesis could constitute an alternative in this population, but the main inconvenience is the high rate of degeneration in this group of patients [6,7]. This study was conducted to assess short-term and medium-term morbidity and mortality after replacement of the aortic valve. What makes this study interesting is that 76% of patients are under the age of 18 and few series in the literature have published on the fate of these patients after this surgery. There is a male predominance as in most series [8]. In a review of the literature, Etnel *et al*. observed an average age of 9.5 years ± 4.9 years for patients benefiting from a replacement of the aortic valve associated with a mitral repair and the rheumatic etiology was less frequent [9].

This was not the case in our series where rheumatic etiology represented the majority of cases. All of our patients have undergone open heart surgery with cardiopulmonary bypass. Operative mortality is zero. The average length of stay at the hospital in the series is 20 days. What has increased the length of stay at the hospital is above all the oral anticoagulant treatment based on antivitamin K (AVK) with the need to recheck the INR (International Normalized Ratio) every 72 hours until you have an effective dose. In other studies, this average length of stay at the hospital was 10 days [10,11]. During the follow-up, only one patient (4%) presented medically treated infectious endocarditis. In the literature lower rates have been found [12-14]. One of the patients (4%) had an accident with anticoagulants such as gingivorrhagia. Minor bleeding complications are mild but should cause AVK to seek an overdose. Even with no bleeding, any AVK overdose puts you at increased risk of bleeding in the next two weeks. It is therefore necessary to quickly restore the INR to a therapeutic area [15]. We did not note during the follow-up a case of reoperations which generally occur after an average age of 7.1 years [8]. The causes of these reoperations after aortic valve surgery are prosthetic mismatch, prosthesis dysfunction, medically uncontrolled endocarditis [16]. Late mortality was 4% (1 death) as in most series in the literature [10,11,17].

## Conclusion

This study reveals that aortic valve replacement surgery, by median sternotomy gives satisfactory short- and medium-term results with negligible morbidity and negligible operative mortality and this technique seems to be safe in this population of young patients. However, these patients require close monitoring and prompt management of complications whether cardiac, infectious, pulmonary, hemorrhagic or renal. Another endpoint is that the valve lesions are so severe in rheumatic pathology that aortic repair is very difficult to perform.

### What is known about this topic

- Etiologies of aortic valve disease are dominated in Africa by rheumatic fever;
- Replacement of the aortic valve is one of the therapeutic strategies for the management of these pathologies;
- It is a surgery with possible complications.

### What this study adds

- Our study shows that the morbidity and mortality in the medium term after aortic valve replacement in young patients is low;
- Our study shows the difficulty to perform aortic repair in this rheumatic context is due to the late diagnosis with severe lesions;
- Our study shows the necessity for rigorous postoperative monitoring for early detection of complications with appropriate management mostly hemorragic complications due to antivitamin K.
